# Prevalence of malaria parasitaemia in school children from two districts of Ghana earmarked for indoor residual spraying: a cross-sectional study

**DOI:** 10.1186/s12936-015-0772-6

**Published:** 2015-06-25

**Authors:** Nimako Sarpong, Ellis Owusu-Dabo, Benno Kreuels, Julius N Fobil, Sylvester Segbaya, Frank Amoyaw, Andreas Hahn, Thomas Kruppa, Jürgen May

**Affiliations:** Kumasi Centre for Collaborative Research in Tropical Medicine, Kumasi, Ghana; German Centre for Infection Research (DZIF), Partner Site Hamburg-Borstel-Lübeck, Hamburg, Germany; Research Group Infectious Disease Epidemiology, Bernhard Nocht Institute for Tropical Medicine, Hamburg, Germany; Section for Tropical Medicine, I. Department of Internal Medicine, University Medical Centre Eppendorf, Hamburg, Germany; Department of Biological, Environmental & Occupational Health Sciences, University of Ghana School of Public Health, Accra, Ghana; AngloGold Ashanti Malaria Control Programme, Obuasi, Ghana

**Keywords:** Malaria prevalence, Asymptomatic parasitaemia, Anaemia, Indoor residual spraying

## Abstract

**Background:**

Indoor residual spraying (IRS) is considered a valuable transmission control measure against malaria but exact efficacy data are not available for many epidemiological settings. This study was conducted to determine indicators for malaria epidemiology and transmission among school children as baseline assessment before IRS implementation in Ghana.

**Methods:**

A cross-sectional study was conducted in Adansi South District of the Ashanti Region and Wa West District of the Upper West Region of Ghana. Malarial parasitaemia and anaemia were determined in pupils between the ages of 2 and 14 years from Early Childhood Development Centres and primary schools. *Plasmodium falciparum* parasitaemia was detected by light microscopy.

**Results:**

Out of 1,649 pupils who were enrolled at participating schools, 684 were positive for plasmodia resulting in a baseline parasitaemia prevalence of 41.5%. Parasite rate was similar in the two districts (42.0% in Adansi South and 40.7% in Wa West), but differed across the nine sentinel schools ranging from 21 to 63% (p < 0.001). The mean haemoglobin concentration was 11.3 g/dl [standard deviation (SD) ±2.1]. Pupils who had moderate to mild anaemia (7.0–10.9 g/dl) constituted 41.7% of the study sample.

**Conclusion:**

The burden of parasitaemia, malaria and anaemia is a major public health problem among school children in rural Ghana with extensive heterogeneity between schools and warrants further investment in intervention measures.

## Background

The World Health Organization (WHO) estimates the burden of malaria to be 198 million cases with 584,000 deaths in the year 2013 being a major cause of poverty and low productivity and vice versa [1]. Despite the fact that decreasing numbers of malaria cases and mortality are recently reported in some endemic regions, most countries in sub-Saharan Africa still suffer from an immense burden of malaria mortality. Data indicates that 90% of global deaths due to malaria occur in Africa, especially among children under age five who are the hardest hit with at least 75% of the fatalities [1].

During the time when the study described here was performed, malaria accounted in Ghana for 32% of all outpatient department (OPD) visits and 49% hospital admissions in children under 5 years of age and morbidity was about three million cases with 4,000 deaths annually [2]. Besides hookworm infections, nutritional deficiencies and haemoglobinopathies, malaria is one of the major causes of anaemia in children [3, 4].

Ghana is implementing a malaria control programme with the aim of reducing malaria morbidity and mortality by 75% by the year 2015 in line with the millennium development goals (MDGs). Indoor residual spraying (IRS) has been introduced among other multiple prevention methods such as insecticide treated bed nets usage and chemoprophylaxis in pregnancy. The IRS programme specifically seeks to cover 90% of all structures in targeted districts [2] and has been conducted by the Anglogold Ashanti Malaria Control Programme (AGAMal) that was implemented as corporate social responsibility in the Obuasi Metropolitan District, Ashanti Region. In the first 2 years of its existence, it attained a 50% reduction in the number of malaria cases in Obuasi, and achieved more than 75% reduction in malaria cases within 6 years of implementation resulting in huge savings on malaria medication expenditure, from $55,000 in 2005 to $6,200 in 2010 (unpublished data). Work absenteeism dropped from 6,983 man-days in 2005 to 163 in 2010 indicating the impact of malaria on health in adults. The programme is currently being scaled up in 40 additional districts in Ghana with funding from the Global Fund.

A malaria parasite prevalence study was conducted at selected sentinel sites in the areas of operation to determine the prevalence of parasitaemia and anaemia among children in sentinel pre- and primary schools as baseline data before the start of the AGAMal IRS programme. In malaria-endemic areas, a significant proportion of children harbours parasites without presenting with signs of clinical malaria [5]. Asymptomatic parasitaemia can nevertheless affect the individuals who carry the parasites and these individuals act as transmission reservoirs [6].

## Methods

### Study setting

This study was conducted in two districts in different Regions in Ghana. The Adansi South District of the Ashanti Region in central Ghana occupies an area of 1,380 km^2^ in the forest zone. Most parts of Ghana are known to be holoendemic for malaria and transmission of parasites is stable in the rainforest area with an estimated entomological inoculation rate (EIR) of >400 per year [7, 8]. The predominant malaria parasite is *Plasmodium falciparum* [6] with an incidence of more than one malaria episode per person year at risk (PYAR) in children under 2 years of age [9]. The Wa West District, covering an area of 1,584 km^2^, is one of nine administrative districts in the Upper West Region in the Northern Belt of of Ghana with almost no detailed data on malaria epidemiology available.

### Sampling procedure and data collection

The data for the study were collected from Early Childhood Development Centres (ECDC) and primary school pupils aged from 2 to 14 years in the period between March and April 2012. Community entry was done by contacting the key stakeholders and informing the community at large through a public address system. Sampling frame was obtained through a list of circuits of schools from the District Education Service Directorate (DESD) in Adansi South District and Wa West District. A multi-stage cluster sampling with probability proportional to size (PPS) was conducted according to the protocol recommended by WHO [10]. Primary clusters were five schools circuits in Adansi South and three circuits in Wa West. A total of nine schools were then sampled from these circuits using simple random sampling with a physical randomization device. All pupils present at these schools at the time of sampling and meeting the inclusion criteria were then enrolled in the study. Whilst not representative for the entire district, these baseline data will allow comparison with the post-intervention data in the same schools.

Of the nine schools selected in the second stage of sampling, six schools were selected in Adansi South (Apagya D/A, Asarekrom D/A, Kokotenten D/A, Kwame Nkyi D/A, New Edubiase D/A, and Nsata Subiriso D/A). The remaining three schools were selected in Wa West (Kpanfa D/A, Lassie–Toulu R/C, and Nyoli R/C) whereas the D/As are public schools and the R/Cs are Roman Catholic schools.

Study participants were randomly selected from all eligible pupils using the schools attendance register as sampling frame. Parents or caretakers and teachers were informed about the background and procedures of the study by the research team. Selected pupils who not rejected to participate were recruited into the study after obtaining informed consent from their parents or caretakers. A standardized questionnaire was administered to the parents, two drops of blood were obtained via finger prick and the tympanic temperature recorded from each participant. Trained research staff interviewed the pupils and completed the questionnaire. An episode of clinical malaria was defined as any asexual *Plasmodium* parasitaemia and a body temperature ≥38°C.

### Laboratory procedures

Thick and thin blood films were prepared from finger-prick blood samples and stained with 10% Giemsa for 30 min and examined by immersion oil microscopy with 1,000× magnification. A slide was declared negative after examining 200 high power fields without parasites. Each slide was read independently and blindly by two certified laboratory microscopists. Parasitaemia was quantified per 200 white blood cells (thick smear) or in case of very high parasitaemia per 1,000 erythrocytes (thin film). Slides with discrepant results were read again by a third reader and the median parasitaemia taken as the final result [11]. The haemoglobin concentration was determined with a HemoCue haemoglobin photometer (HemoCue AB, Angelholm, Sweden) as stated by the manufacturer using capillary blood samples from finger pricks.

### Statistical analysis

Data were double-entered and cleaned by trained data entry staff at the KCCR using MS Access 2010 and then imported into STATA version 12 (Stata Corp., College Station, Texas: StataCorp LP, USA) for statistical analyses. Proportions are presented as descriptive statistics for all categorical variables. Prevalence of parasitaemia was calculated as overall prevalence and separately for each district. 95% confidence intervals (95% CI) were estimated to provide uncertainty surrounding the point estimates. Means and standard deviations for haemoglobin levels were calculated as overall mean and separately by district and by parasitaemia status. Anaemia was defined as follows: Hb level 10.9–7 g/dl (moderate anaemia) and <7 g/dl (severe anaemia). The level of statistical evidence for an association between parasitaemia and personal characteristics adjusted for school was assessed in a univariate conditional logistic regression. Variables that showed some evidence of an association with parasitemia (p < 0.10) were then included in a multivariate conditional regression model adjusted for school to account for potential confounding. Adjustment for district in univariate and multivariate analyses was not performed as there was no variation of this parameter after adjustment for school. Statistical significance was set at a default alpha of 0.05.

### Ethics

The study was conducted in accordance with the ethical principles of the Declaration of Helsinki. Ethical approval for the study was obtained from the Ethics committees of the School of Medical Sciences, Kwame Nkrumah University of Science and Technology (KNUST). Administrative clearance was obtained from district directorates of Ghana Education Service.

## Results

A total of 1,649 pupils, 988 from Adansi South District and 661 from Wa West District, were enrolled in the study from nine schools. The ages of the pupils ranged from 2 to 14 years with a median age of 10 years. Some personal characteristics of study participants varied significantly between the two districts (Table 1). Participants in Adansi South were slightly older compared to participants from Wa West (median 9.7 vs 9.3 years). In Adansi South compound houses (small areas tightly surrounded by little connected buildings with a central court) were more common (19.0% vs 4.6%) and reported bednet use was higher (56.2% vs 31.6%). Finally, participants from Adansi South were less likely to be enrolled in the  National Health Insurance Scheme (NHIS) (44.9% vs 56.7%) and also sought care at a hospital less frequently if taken ill (49.9% vs 58.0%).

**Table 1:** Characteristics of study participants stratified by district

	District				Total^a^ (N = 1649)		p value^c^
	Adansi South (N = 988)		Wa West (N = 661)				
	N	%^b^	N	%^b^	N	%^b^	
Sex							
Female	520	52.6	296	44.8	821	49.6	0.002
Male	468	47.4	365	55.2	833	50.4	
Age, years (SD)	988	Mean 9.7 (±2.7)	661	Mean 9.3 (±2.9)	1649	Mean 9.5 (±2.8)	0.01
Hospital if sick^d^							
No	493	50.2	274	42.0	767	46.9	0.001
Yes	489	49.8	378	58.0	867	53.1	
House type^e^							
Compound	186	19.1	29	4.6	215	13.3	<0.001
Semi-detached	143	14.7	81	12.7	224	13.9	
Single	645	66.2	527	82.7	1,172	72.8	
Bed net use^f^							
No	433	43.8	446	68.4	879	53.6	<0.001
Yes	555	56.2	206	31.6	761	46.4	
NHIS enrolment^g^							
No	542	55.0	286	43.3	828	50.4	0.002
Yes	443	45.0	374	56.7	817	49.6	

*SD* standard deviation.

^a^If numbers do not add up to 1,649 in any category, this is due to missing values in the respective field. (Missing values in each category: Adansi South/Wa West; sex 0/0; age 0/0; hospital if sick 6/9; house type 14/24; bed net use 0/9; NHIS enrolment 3/1).

^b^Mean for age.

^c^Chi squared test for heterogeneity for categorical variables. Student’s *t* test for difference of means for age.

^d^Individuals who reported to visit a hospital if sick according to the interview; 15 missings.

^e^38 missing.

^f^Bed net use: if a pupil slept under a bed net in the night before the survey; 9 missing.

^g^NHIS National Health Insurance Scheme; 4 missing.

### Parasitaemia prevalence and Plasmodium species

The overall parasitaemia prevalence in the study sample was 41.7% and was similar in the two districts: 42.0% [95% confidence interval (CI) 38.9–45.1] in Adansi South and 40.7% (95% CI 36.9–44.4) in Wa West (Table 2). However, the proportion of participants with parasitaemia showed strong heterogeneity between different schools in Adansi South (p < 0.001), varying from 21% in New Edubiase to 63% in Nsata Subiriso (Table 2). Species of *Plasmodium* detected were: single infections with *P. falciparum* in 95.9%, *Plasmodium malariae* in 2.3%, and *Plasmodium ovale* 0.2% of all cases of parasitaemia. Mixed infections with *P. falciparum* and *P. malariae* were present in 1.6% of all cases. Gametocytes were present in 7.6% of participants with parasitaemia (3.1% of all participants).

**Table 2:** Prevalence of parasitaemia and anaemia stratified by schools and districts

	Parasitaemia				Haemoglobin					
	No		Yes		≥11 g/dl		10.9–7 g/dl		<7 g/dl	
	N	%	N	%	N	%	N	%	N	%
Adansi South^a^										
Apagya	176	72.1	68	27.9	136	57.1	89	37.4	13	5.5
Asarekrom	64	38.1	104	61.9	62	36.9	103	61.3	3	1.8
Kokotenten	50	37.6	83	62.4	40	30.1	87	65.4	6	4.5
Kwame Nkyi	32	47.1	36	52.9	20	29.4	47	69.1	1	1.5
New Edubiase	211	79.0	56	21.0	148	55.4	108	40.5	11	4.1
Nsata Subiriso	40	37.0	68	63.0	20	18.5	82	75.9	6	5.6
Total	573	58.0	415	42.0	426	43.4	516	52.4	40	4.1
Wa West^a^										
Kpanfa	159	74.7	54	25.3	152	72.0	59	28.0	0	0
Lassie Tuolu	117	48.0	127	52.0	162	66.9	80	33.1	0	0
Nyoli	116	56.9	88	43.1	174	86.6	27	13.4	0	0
Total	392	59.3	269	40.7	488	74.6	166	25.4	0	0

^a^Chi squared test for heterogeneity for parasitaemia and haemoglobin: Adansi South, p < 0.0001; Wa West, p < 0.001; overall p < 0.001.

### Haemoglobin concentration

The mean haemoglobin concentration in the study population was 11.3 g/dl (SD ±2.1) (Table 2) and differed slightly between the two districts [Adansi South: 10.9 g/dl (SD ±2.5); Wa West: 11.8 g/dl (SD ±1.3); p < 0.001]. Overall, 55.9% (n = 914) had a normal Hb level (Hb ≥ 11.0 g/dl), 41.7% (n = 682) had a mild to moderate anaemia (Hb 10.9–7 g/dl) and 2.4% (n = 40) a severe anaemia (Hb < 7.0 g/dl). The prevalence of anaemia differed considerably  between the two districts [mild to moderate anaemia: Adansi South 52.5% vs Wa West 25.4% (Table 2)].

### Body temperature

Body temperature of the pupils ranged from 36.0 to 39.4°C with a mean of 37.3°C (SD ±0.38). Out of the 1,649 apparently healthy participants attending school, 23 (1.4%) had a temperature ≥38.0°C. Out of these, 11 (0.6%) were positive for parasitaemia and thus fulfilled the case definition of symptomatic malaria and of which nine came from the Adansi South. The cases were equally distributed between the schools.

### Parasitaemia and anaemia

The mean haemoglobin concentration was significantly lower (p < 0.001) in participants with parasitaemia (10.9 g/dl, SD ±2.1) compared to those without (11.6 g/dl, SD ±2.1). Corresponding to this, only 47.6% of parasitaemic participants had a normal Hb compared to 61.8%   of the group without parasitaemia  (p < 0.001).

### Association between personal characteristics and parasitaemia

Several of the surveyed personal characteristics were associated with a risk of parasitaemia. Table 3 shows the unadjusted (univariate) and adjusted (multivariate conditional logistic regression) analyses of the influence of personal characteristics on the odds of parasitaemia. There was declining risk for parasitaemia with age (multivariate: OR 0.91, 95% CI 0.87–0.95, p < 0.001) and NHIS enrolment (multivariate: OR 0.75, 95% CI 0.60–0.94, p = 0.01). There was no evidence for an association between housing type and parasitaemia. Both the univariate and multivariate analysis indicated that having slept under a bed net in the night before the survey was associated with a higher risk of parasitaemia, but this trend was not significant in the multivariate analaysis (univariate: OR 1.28, 95% CI 1.02–1.59, p = 0.03; multivariate: OR 1.25, 95% CI 0.99–1.57, p = 0.06).

**Table 3:** Conditional logistic regression (adjusted for school) of personal characteristics

	Parasitaemia						
	Postives, in %	Univariate			Multivariate		
	n = 1,649	OR	95% CI	p value	OR	95% CI	p value
Sex							
Female	39.0	1			1		
Male	43.9	1.21	0.98–1.49	0.08	1.20	0.98–1.49	0.09
Age (per year)^a^		0.91	0.87–0.94	<0.001	0.91	0.87–0.95	<0.001
Hospital if sick^b^							
No	42.6	1					
Yes	40.7	0.88	0.71–1.09	0.24			
House type^c^							
Compound	28.4	1					
Semi-detached	45.1	1.18	0.77–1.83				
Single	43.2	1.14	0.80–1.62	0.73			
Bed net use^d^							
No	35.6	1			1		
Yes	48.1	1.28	1.02–1.59	0.03	1.25	1–1.56	0.06
NHIS enrolment^e^							
No	45.5	1			1		
Yes	37.3	0.77	0.62–0.96	0.02	0.75	0.60–0.94	0.01

*OR* odds ratio, *CI* confidence interval.

^a^Linear regression.

^b^Individuals who reported to visit a hospital if sick according to the interview; 15 missings.

^c^38 missings.

^d^Individuals who reported to use bed nets regularly according to the interview; 9 missings.

^e^NHIS, member of National Health Insurance Scheme; 4 missings.

## Discussion

Prevalence of malaria parasitaemia among school children without obvious illness was 42% and 41% in participating schools within Adansi South and Wa West, respectively. While the overall parasite rate in the two districts was similar, there was a strong variation of parasitaemia prevalences across schools. As a tendency, the frequency of parasite carriers without fever was higher in the remote villages (e.g. Nsata Subiriso, 63%) than in the district capital (New Edubiase, 21%). This finding is in accordance with other studies demonstrating that the most exposed households are those in the more remote parts of a village or district [12]. Microscopic detection of parasites as used in the study presented here may be limited by a low sensitivity in comparison to PCR and information on multiplicity of *P. falciparum* infection is lacking. Nevertheless, microscopy was considered sufficient for the comparison of parasite prevalences before and after IRS intervention given that the same methodology is used.

Notably, 11 of the study participants attending school fulfilled the WHO criteria of malaria. The occurrence of malaria in children at school is in line with findings from other countries in sub-Saharan Africa [13, 14] with major impact on performance [15]. The observation that there was no association between malarial parasitaemia and tympanic temperature or fever is in contrast to the findings reported in another study from Cameroon were a significant association between axillary temperature and malarial parasitaemia among apparently asymptomatic school children was found [16]. The observation in the current study could possibly be due to the low number of participants with fever (n = 23) and the resulting low statistical power for this question. Children with *Plasmodium* infection might have been co-infected with other pathogens causing the fever and resulting in a misclassification as malaria [17].

Similar to the prevalence of parasitaemia, haemoglobin levels and the prevalence of anaemia varied widely between schools. However, there was also significant variation between the two districts because, all cases of anaemia with a haemoglobin <7 g/dl were detected in Adansi South. Interestingly, 9 of the 11 children with fever came from one village possibly indicating a small outbreak of another infection. Furthermore, while our data showed that parasitaemia and anaemia were associated, the different prevalence of anaemia in two districts with similar prevalence of parasitaemia indicated that other co-factors might be involved. Previous studies have shown that causes of paediatric anaemia beyond malaria in Ghana include various nutritional deficiencies, other infections, as well as genetic defects including haemoglobinopathies [18].

Several personal characteristics of the participants were associated with a risk for parasitaemia. As expected, the odds of parasitaemia declined with age. In stable transmission areas such as in Ghana, parasitaemia and malaria symptoms are strongly dependent on the development of semi-immunity after prior exposures [19].

Surprisingly, the data showed that reported use of a bed net in the night before the survey was not associated with any protective effect but, as a tendency, was associated with a slightly higher risk of infection. This finding may be by chance or a result that is confounded by transmission heterogeneity on a microepidemiological scale [20]. Living in an area of high transmission may lead to a higher rate of net usage due to the nuisance of mosquito bites while individuals living in lower transmission areas may not use nets that often. This could result in the paradoxical finding that net usage is a risk factor for parasitaemia. Moreover, there are no data on the quality of the nets, correct use, or whether they were treated with insecticides.

As anticipated, parasitaemia prevalence among pupils enrolled in the national health insurance scheme was lower than those who were not enrolled. Possibly those with membership in NHIS might have better chance of seeking medical care compared to those without membership. It has been shown that households participating in the NHIS scheme had a higher socio-economic status [21] and children from these families had a lower risk for malaria.

## Conclusions

The heterogeneity of parasitaemia and haemoglobin levels was high among schools. Accordingly, a large number of schools must be monitored to provide representative figures for a district. The present data will serve as pre-intervention data for comparison with post-intervention data for the estimation of IRS efficacy. It is recommended to perform regular evaluations over time since the degree of temporal and seasonal fluctuations of malaria and haematological parameters in the study areas have neither been investigated previously nor in the present study. The findings indicate that the burden of malaria and anaemia is a relevant public health problem among school children in Ghana and warrants investment in intervention measures to stem the tide.
